# Upper Palaeolithic fishing techniques: Insights from the engraved plaquettes of the Magdalenian site of Gönnersdorf, Germany

**DOI:** 10.1371/journal.pone.0311302

**Published:** 2024-11-06

**Authors:** Jérôme Robitaille, Lisa-Elen Meyering, Sabine Gaudzinski-Windheuser, Paul Pettitt, Olaf Jöris, Robert Kentridge

**Affiliations:** 1 MONREPOS, LEIZA, Neuwied, Germany; 2 Durham University, Durham, United Kingdom; 3 Johannes Gutenberg-University Mainz, Mainz, Germany; Universita degli Studi di Ferrara, ITALY

## Abstract

The ~15,800 year-old Magdalenian site of Gönnersdorf, in Germany, has produced 406 engraved schist plaquettes which have been extensively studied in the past. The introduction of advanced imaging technologies, notably Reflectance Transformation Imaging (RTI), has now precipitated a re-evaluation of these artifacts, uncovering nuanced depictions of fishing practices previously unrecorded for the Upper Palaeolithic. Our investigation harnesses RTI to elucidate fine engraving details on the plaquettes, revealing depictions of fish and accompanying grid motifs. The analytical process enabled by RTI has exposed an intricate link between the grid patterns and fish figures, showing that they were a deliberate combination portraying the use of fishing nets. This discovery posits a significant departure from earlier interpretations of the site’s iconography, which predominantly emphasized more naturalistic representations of fauna. Furthermore, these findings illuminate aspects of Magdalenian cultural praxis, suggesting that representations of aquatic life and fishing technologies were not merely utilitarian in nature but were embedded within a broader symbolic framework. This study enhances our comprehension of Magdalenian peoples’ interaction with the aqueous milieu, revealing a sophisticated symbiosis between ecological adaptation and artistic expression.

## Introduction and background of research

The exploitation of aquatic resources has a deep antiquity [1, 2]. It is widely accepted that freshwater fishing was regularly practised throughout the European Upper Palaeolithic, as evidenced by the discovery of fish remains at several archaeological sites [3–6]. Zooarchaeological data suggest that fish consumption increased during the second half of the Upper Palaeolithic [7–11]by which time it appears to have become a relatively common food source, especially during the Late Upper Palaeolithic Magdalenian period (~19,000–13,000 cal. BP [12]. Prehistoric fishing deployed a diverse range of technologies and techniques, including various strategies for obtaining fish that can be summarised as *active fishing*, which requires direct human involvement with or near the fishing equipment, *passive fishing*, involving trapping methods [1], or a combination of both. Some methods were developed for solitary (individual) catches, such as angling, while others were refined to maximize yields through the use of collective nets and traps. The selection of a particular method was influenced by the target fish species, as well as the distinct habitats and terrains where specific aquatic resources thrived.

Although evidence for fishing in the Upper Palaeolithic is not abundant, there is sporadic direct and indirect evidence of several fishing techniques, such as barbed points or harpoons, bows and arrows (all of which can be used additionally to procure terrestrial game), traps, and fishing nets. Barbed points were a Magdalenian innovation which significantly improved the hunting of small game, fish, and birds [13, 14]. Morales-Muñiz (2010) [15] noted that an increasing use of spears to obtain aquatic targets was probably responsible for the development of the harpoon, accompanied with a detachable head connected to a line for improved retrieval Weniger [16–18] categorized Magdalenian barbed points into four functional types, all probably related to fishing, a connection further supported by ethnographic analogies [14, 19]. Bow and arrow fishing was likely limited to still, freshwater environments [20], but its effectiveness was reduced in moving water due to the arrow’s small mass and the water’s viscosity [15]. The earliest archaeological objects interpreted as fishhooks may be as old as 42,000 years, i.e. the Early Upper Palaeolithic [21], in Europe, demonstrable fishhooks are not known before the Magdalenian [22–24]. Additionally, they were versatile: while primarily used for fishing, some evidence suggests they may have been used for bird hunting [25]. Traps and weirs, made from perishable materials, were likely though used, although archaeological evidence is limited [15, 19, 20].

Numerous depictions of fish appear in Upper Palaeolithic—particularly Magdalenian–parietal and portable art [26]. Despite the representation of fish, representations of fishing activities remain very rare, and mainly feature harpoons, arrows, trapping, and fishhooks [1, 26]. A fishing scene ("*une scène de pêche*,") was engraved on the wall of Los Casares cave, Spain, where the imagery includes 22 or 23 anthropomorphic figures, associated with a remarkable painting featuring 16 depictions of fish with a human diver prominently placed on the panel’s far left [27]. The right-hand side of this composition reveals a diverse range of stylistic techniques in the fish representations, from simple lines to intricate patterns. Some fish exhibit finely detailed fins and eyes, and at least two appear to be impaled by spears/harpoons. In Magdalenian art, depictions of fish with arrows or adorned with line patterns that suggest harpoons or arrows occur relatively frequently. For example, in La Grotte des Combarelles I (Les Eyzies, Dordogne), Barrière (1983) identified a schematic depiction of an oval-shaped fish (no.70 in his classification), with a distinctive dorsal extension, reminiscent of a harpoon or arrow [28]. Additionally, in the same cave, (in Composition 68), a pattern of lines is suggestive of harpoons or arrows [28: 329, Fig 340 VIIIG952] [29: 86, Fig 3]. In the Las Grajas cave network, Panel 56 features a fish representation, apparently pierced by an arrow or assegai [29–32: 116, Fig 17]. Nearby, on a pillar, an engraved fish, overscored with black lines and pierced by an arrow or harpoon, evokes a flatfish [29: 116, Fig 17]. Other figures representing fish pierced by assegais, arrows, or harpoons have been observed in various French and Spanish caves including Altamira, Los Casares, Nerja, Romanelli, Niaux, Ekain, and Monedas [27, 29, 31, 33–42]. By contrast, only a few depictions of fishing nets or traps are known, at least with any confidence. In La Grotte des Combarelles I (Les Eyzies, Dordogne), an engraving of a fish with its head hidden in a geometric "trap" made of intersecting parallel lines spans 40 cm [28, 29: 84–86, Fig 3]. Additionally, a scene dated to the Late Pleistocene-Early Holocene period features a fish-shaped figure measuring 74x10 cm, with its head and tail extending beyond a rectangular grid measuring 37.5x24 cm. The fish, marked by peckings that may represent an eye, has a second ventral line emphasizing its head. The overlapping grid suggests a unified scene, probably fishing [43, 44: Fig 8]

Depictions of fish and fishing, although less common in *cave* art compared to terrestrial animals like horses, cattle, deer, and goats, are more frequently found in *portable* art forms, as noted by Cleyet-Merle (1987) [45]. An engraved bone from Laugerie-Basse—the "*La Pêche Miraculeuse*" (Miraculous Fishing Scene)—depicts an anthropomorphic figure bearing a disproportionately elongated arm ending in a stylized hand, apparently reaching for a large salmonid [29: 109, Fig 14]. In the Labastide cave (Hautes-Pyrénées), a fish depicted on a schist slab is overlain by eleven vertically oriented arrows. Similarly, in Gourdan cave (Haute-Garonne), a fish is depicted on a pebble, with three symbols resembling arrows intricately engraved onto its body [26]. Only two notable instances of Palaeolithic depictions of angling can currently be identified with any confidence. The first, identified by Bottet and Bottet in 1949 [46], is a pebble from La Baume-Bonne, Quinson (Basses-Alpes), is likely a salmonid (i.e. trout or salmon) indicated by an elongated head, spindle-shaped body, and the precise shape and positioning of its fins. Near the fish’s caudal fin, there is a smaller fish with a thin, forked tail. An obliquely ascending line is attached to a point of this smaller fish, which therefore seems to represent bait whether dead or alive [46: 266, Plate III, No. 10]. This depiction strongly suggests an angling scene, and similar—if less clear—representations on other pebbles have been noted, such as engraving No. 9 in Plate II and No. 14 in Plate III [46]. The second instance, described by Abbé Breuil in 1908 from the site of Bruniquel (Pyrénées), is found on reindeer antler. Breuil’s study titled: "*Les petits instruments magdaléniens à pointe bifide ou tridentée de Bruniquel et quelques autres gisements*" [47] discussed a scene where a fish is depicted with a device in front of its mouth featuring two recurring barbs. This device is interpreted as a hook, upon which the fish is likely to bite, rather than as a point of a harpoon [47: 190, Fig 9]. A depiction of a fishing trap is found at La Baume-Bonne. Here, a large oval pebble of grey limestone displays a highly stylized fish on one edge, complete with distinct features such as its caudal fin. Small, evenly spaced transverse strokes surround the fish. On the opposite edge of the pebble, a larger, enigmatic motif emerges, resembling a device for capturing fish, similar to a fish trap or a nasse [46: 266 Plate III Fig 10a, 10b]. Notably, there is a lack of artistic representation of net fishing, a technique likely used, given the archaeological evidence from the same period.

The infrequent depiction of fish in parietal art does not of course undermine their importance in Upper Palaeolithic diet. As Dams (1987) suggested, the relative ease (and safety) of catching fish compared to larger prey may have rendered them less ‘impressive’ artistic subjects, although stable isotope data from human remains on European Upper Palaeolithic sites suggest that from the Mid (and probably from the Early) Upper Palaeolithic aquatic resources were contributing up to 50% of dietary protein [48]. Leroi-Gourhan (1965) also hypothesized that the rarity or absence of an animal in art might actually indicate its relative commonplace presence in the daily diet, or conversely, its significant status within the community. The most commonly depicted fish in Magdalenian art are salmonids, clearly identifiable due to their distinctive features, and occasionally, these possess sufficient detail for species identification [1, 26]. Palaeolithic artists occasionally depicted a variety of other species, however, including pike, sturgeon, eel, cyprinids, and flatfish. Even marine species such as alosa and lamprey were possibly portrayed, although these identifications are more speculative [26, 49].

Here, we provide new evidence of the depiction of fishing activities from the Central European Late Magdalenian, which clearly depict fishing technologies that have been previously unrecognized in this period. The rarity of such depictions presents interpretative challenges, yet, offer invaluable insights into the fishing practices of the time. We leverage advanced imaging technology such as RTI to examine the engraved schist plaquettes from the ~15,800-year-old Late Magdalenian site of Gönnersdorf on the northern bank of the river Rhine in western central Germany [50–53].

## Methodology

We employed a comprehensive methodological approach to investigate Upper Palaeolithic fishing practices, as depicted in the engravings on the Gönnersdorf schist plaquettes. This is a collection of 406 engraved plaquettes characterized by their unique artistic styles and iconographic themes depicting both terrestrial and aquatic animals [53–55] and humans [53, 56, 57], and it is curated at the MONREPOS Archaeological Research Centre and Museum for Human Behavioural Evolution, in Germany.

Reflectance Transformation Imaging (RTI) was systematically applied to the entire corpus of engraved plaquettes. RTI technology applications have recently opened new avenues in the study of prehistoric art and traceology [58, 59]. This advanced imaging technique was instrumental in capturing high-resolution, detailed images of the plaquettes, facilitating a nuanced examination of their engravings, and was essential in our work for its ability to reveal fine details and textures. RTI’s capability to manipulate light and shadow in a digital environment allowed us to accentuate subtle details on the engraved surfaces, revealing aspects that traditional observation methods might overlook. Traditional methods, such as direct visual inspection under static lighting conditions or simple photographic documentation, often fail to capture the full depth and intricacy of engravings due to their limited ability to enhance surface topography and texture. By contrast, RTI, and equipment such as the RTI Dome fitted with controlled lighting and high-resolution cameras, can produce images with variable lighting angles, including enhanced magnification capabilities [60].

The use of RTI technology in the analysis of Gönnersdorf plaquettes was instrumental in the identification of fishing practice. The ‘Specular Enhancement’ mode in particular allowed us to identify engraved lines that cannot otherwise be seen under normal lighting conditions. RTI enabled us to illuminate the engraved surfaces from various angles, revealing fine lines and subtleties that are invisible under standard, static lighting conditions. Through this process, we could detect engraving nuances with a precision that traditional methods could not achieve. The ability to manipulate the light source digitally allowed us to highlight specific features, such as the angles at which the engravings were made, providing insights into the tools and techniques used by the original artists. Moreover, the stratigraphy of intersecting and overlapping engraved lines became much clearer, allowing us to determine the sequence in which the lines were carved. This was crucial in understanding the layering and development of the engraved motifs. Furthermore, we were able to recognize connecting engraved lines, and thus identify compositions of fusiform and grid motifs. These insights were instrumental in advancing our understanding of the artistic practices and symbolic expressions of the people who created these artifacts.

## Description of the evidence from Gönnersdorf

A total of eleven depictions of fish were identified among the Gönnersdorf engravings, of which only four had previously been recognized: fish in Plaquettes 213, 280, 281 and 282 were published by Bosinski [54: 129 plate 146–149]. With the systematic application of RTI we identified an additional seven plaquettes (341, 346, 347, 355, 369, 374, and 402) featuring fusiform or fish shapes. The fish in Plaquette 282, as well as all seven newly identified fish are depicted in association with lines arranged in grid-like patterns. We therefore have eight plaquettes (282, 341, 346, 347, 355, 369, 374, and 402) which depict the fish- and -grid motif, which all share the following physical and stylistic attributes:

### Plaquette size and dimensions

All eight plaquettes are compact in size, with dimensions ranging from 6 cm in length by 5.4 cm in width, to 13.5 cm in length by 12.5 cm in width. Their thickness varies from 0.5 cm to 2.1 cm (Table 1).

**Table 1:** Integrity and dimensions of Gönnersdorf fish-and-grid plaquettes.

Plaquette number	Integrity of the plaquette	Plaquette dimensions (cm)		
		Length	Width	Thickness
Gö 282 (Gö 9:. d 117)	Engraved on a fragment	9.5	7.5	1.4
Gö 341	Complete	13.5	6.5	1.4
Gö 346	Complete	11.5	9.3	1.4
Gö 347 (Gö 350:’ Pl.I 33)	Engraved on a fragment	6	7	1
Gö 355	Complete	9.7	8.5	1.4
Gö 369	Complete	7.6	5.4	0.8
Gö 374	Complete	11	12.5	1.6
Gö 402	Complete	11	11	2.1

This table presents the integrity and dimensions of Gönnersdorf plaquettes bearing the Fish-and-grid motif, specifying their length, width, and height measurements.

### Surface texture

**Fig 1:**
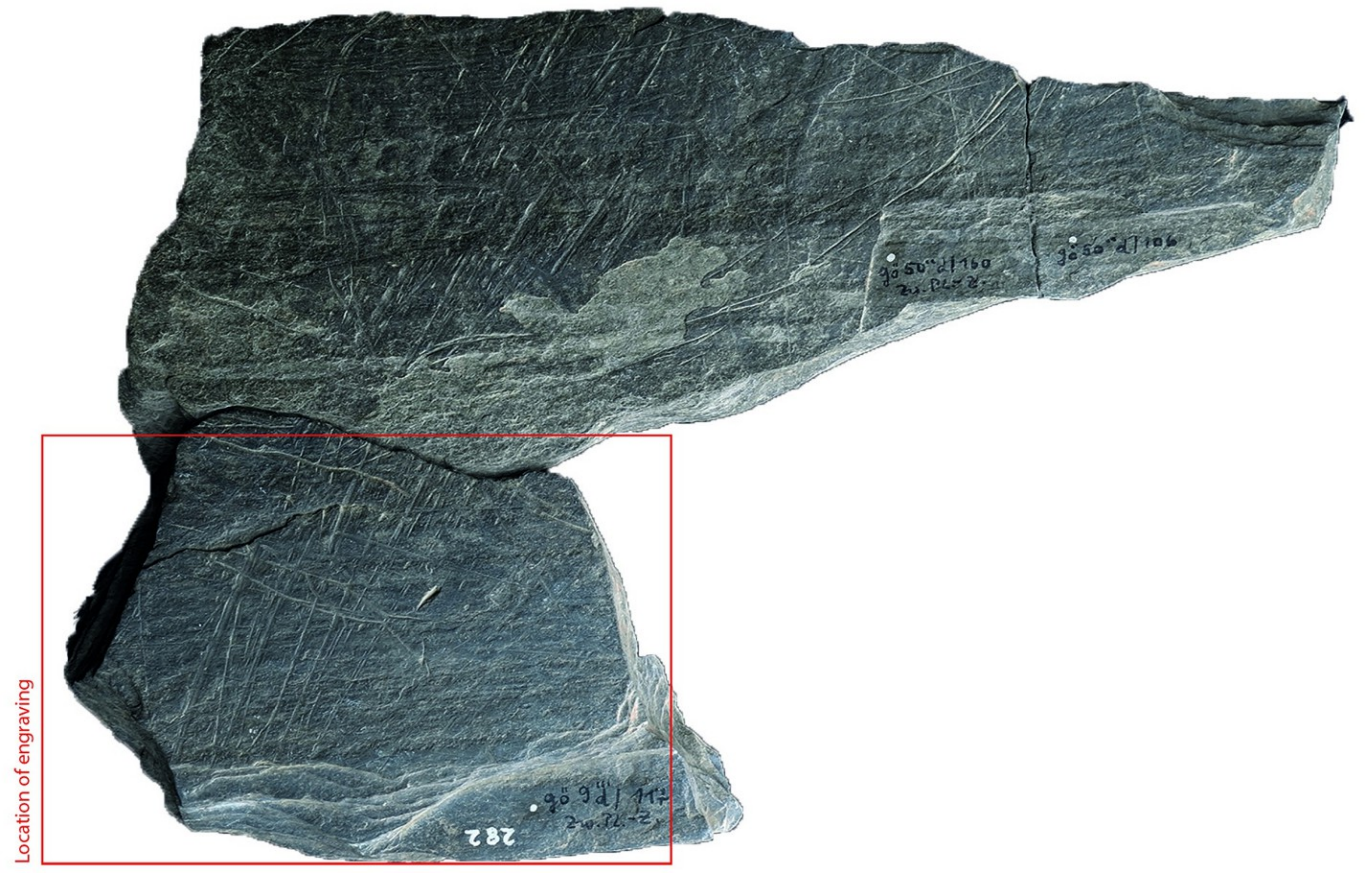
Plaquette 282, Gl 9:. d 117; Gö 50: d 160; Gö 50: d 106. Dimensions of plaquette: 18cm (L) x 14.3cm (W) x 1.4cm (T); Dimension of fragment (Gö 9:. d 117): 9.5cm (L) x 7.5cm (W) x 1.4cm (T).

**Fig 2:**
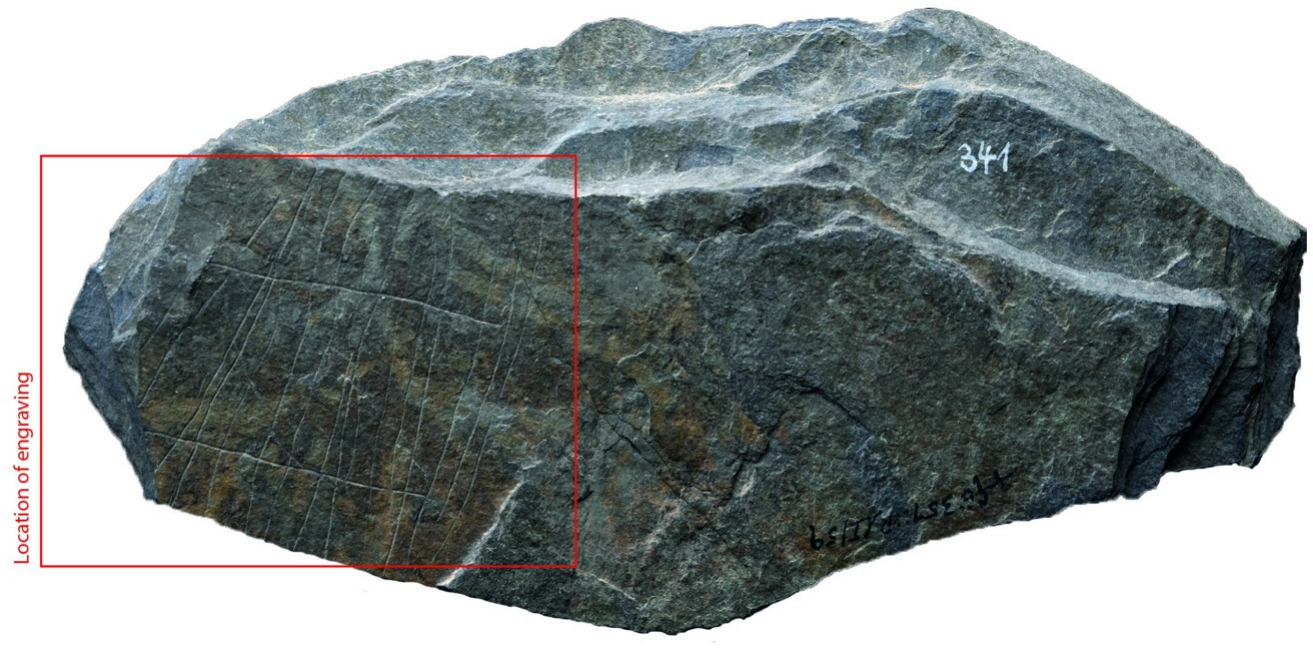
Plaquette 341, Gö 351::Pl.I/59. Dimensions of plaquette: 13.5cm (L) x 6.5cm (W) x 1.4cm (T).

**Fig 3:**
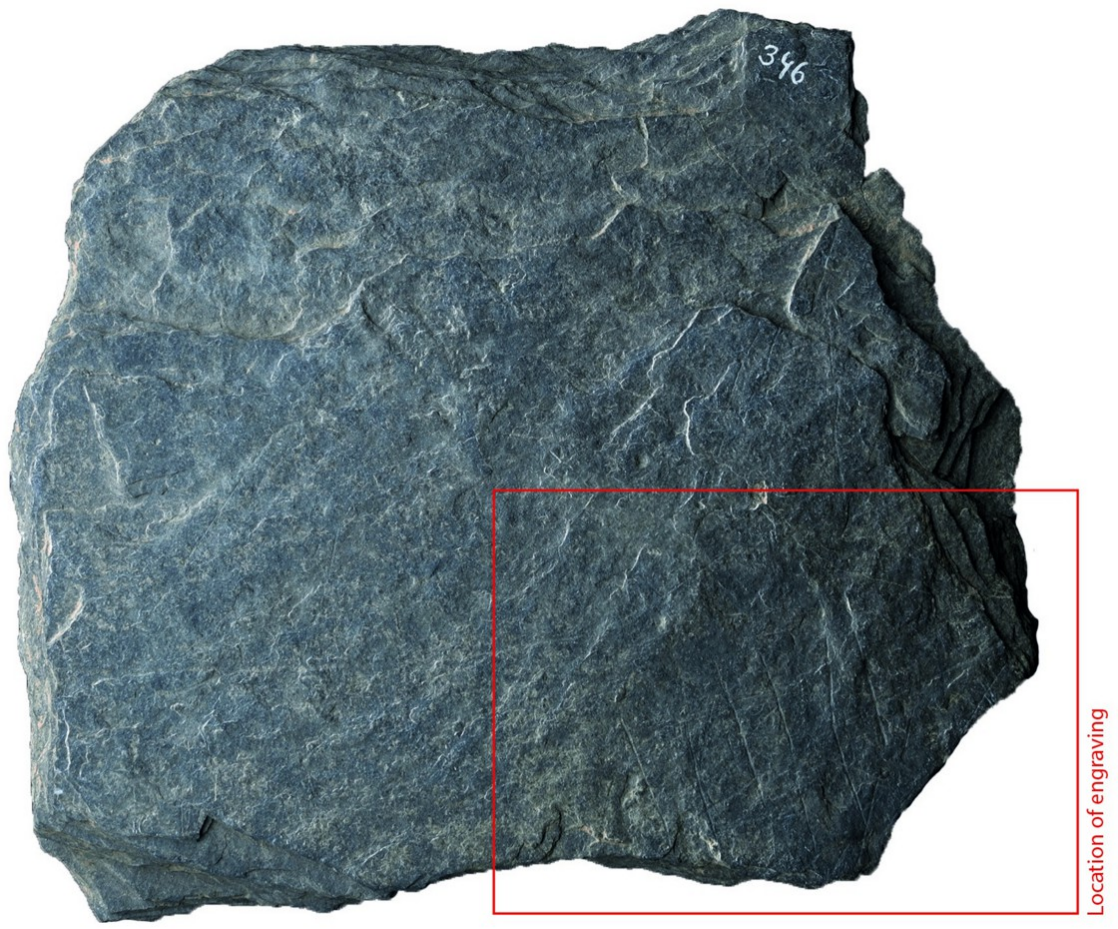
Plaquette 346, Gö 69’ c/46 Pl.I. Dimensions of plaquette: 11.5cm (L) x 9.3cm (W) x 1.4cm (T).

**Fig 4:**
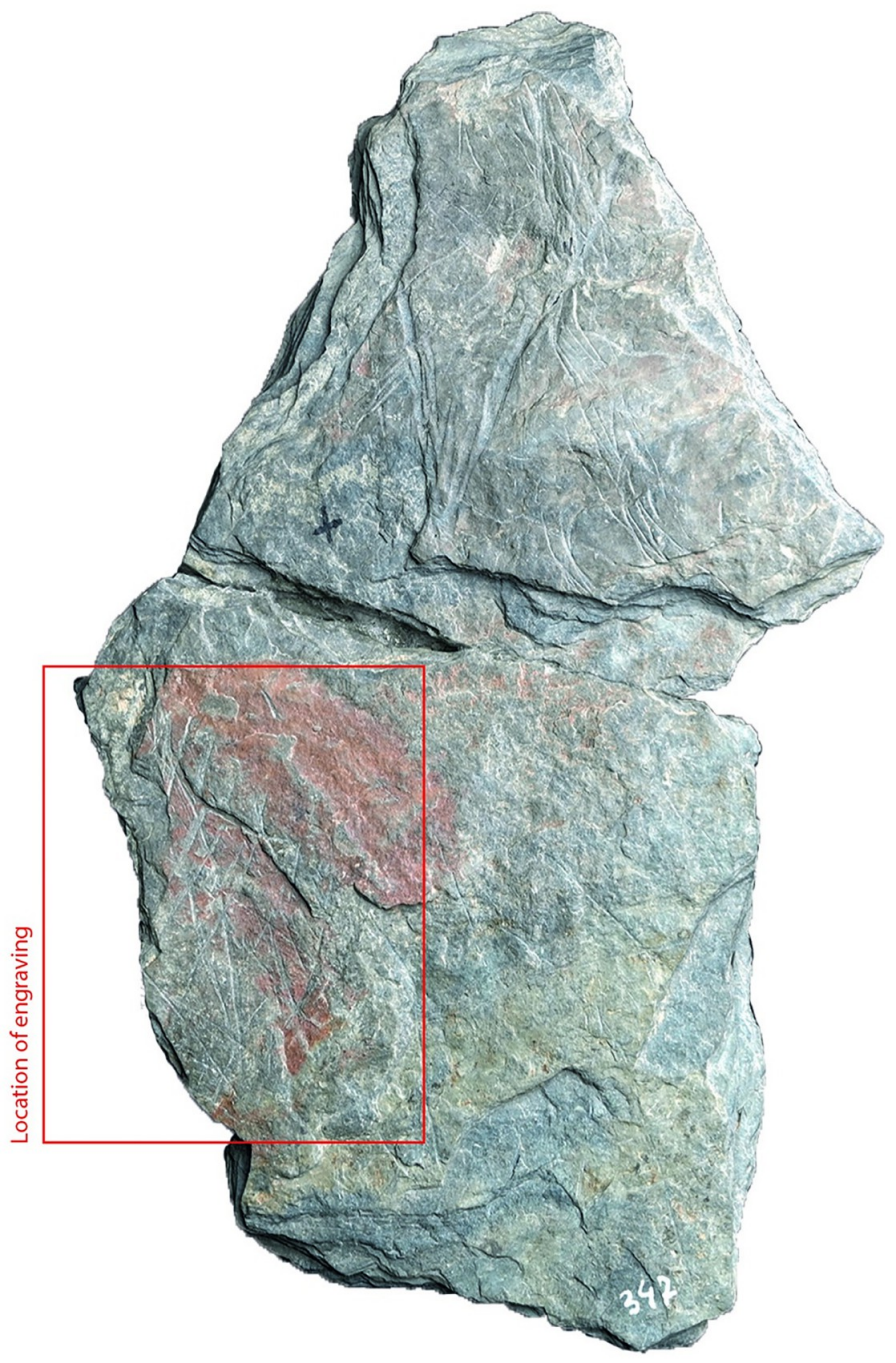
Plaquette 347, Gö 350:’ Pl.I 33; Gö 350:’ Pl.I 30. Dimensions of plaquette: 12.2cm (L) x 8.5cm (W) x 1cm (T); Dimension of fragment (Gö 350:’ Pl.I 33): 6cm (L) x 7cm (W) x 1cm (T).

**Fig 5:**
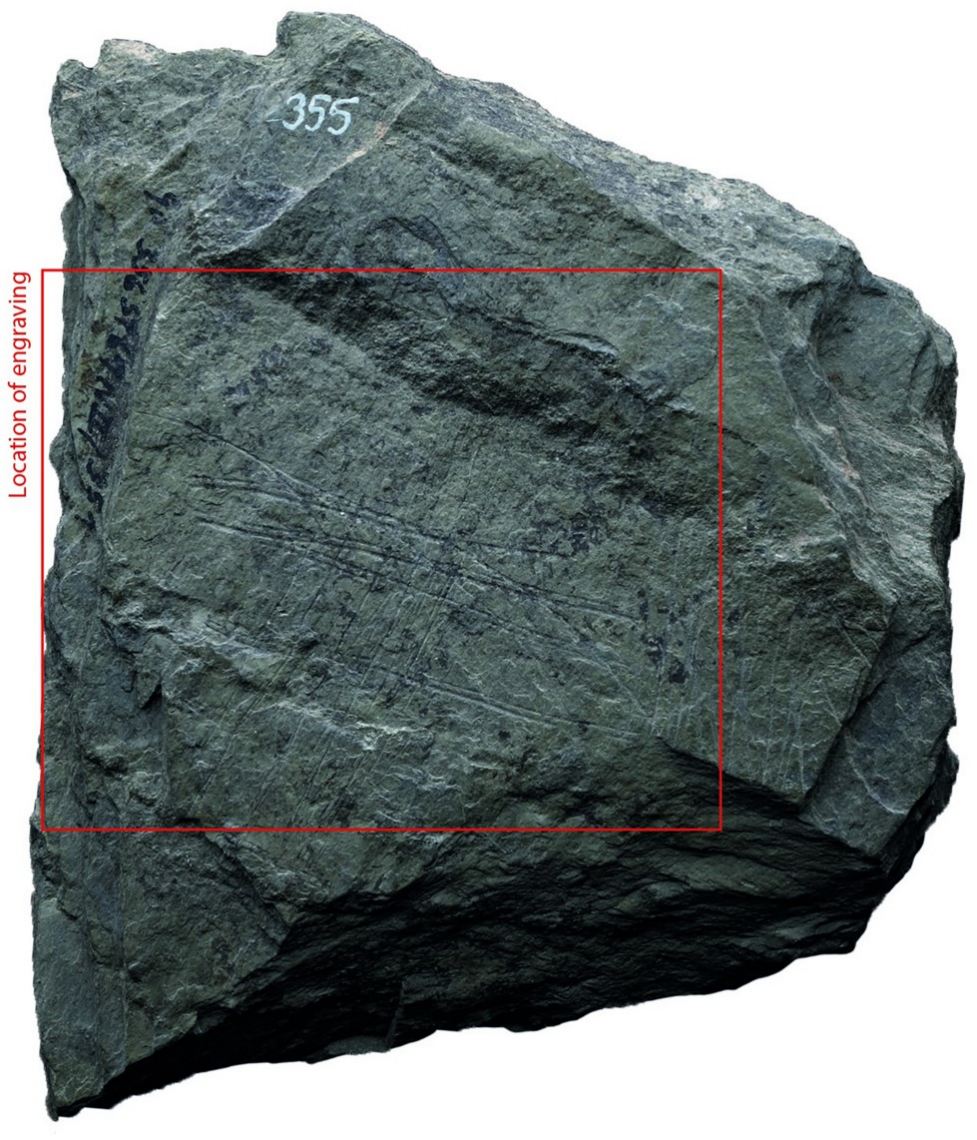
Plaquette 355, Gö 356 St67 N.II/351. Dimensions of plaquette: 9.7cm (L) x 8.5cm (W) x 1.4cm (T).

**Fig 6:**
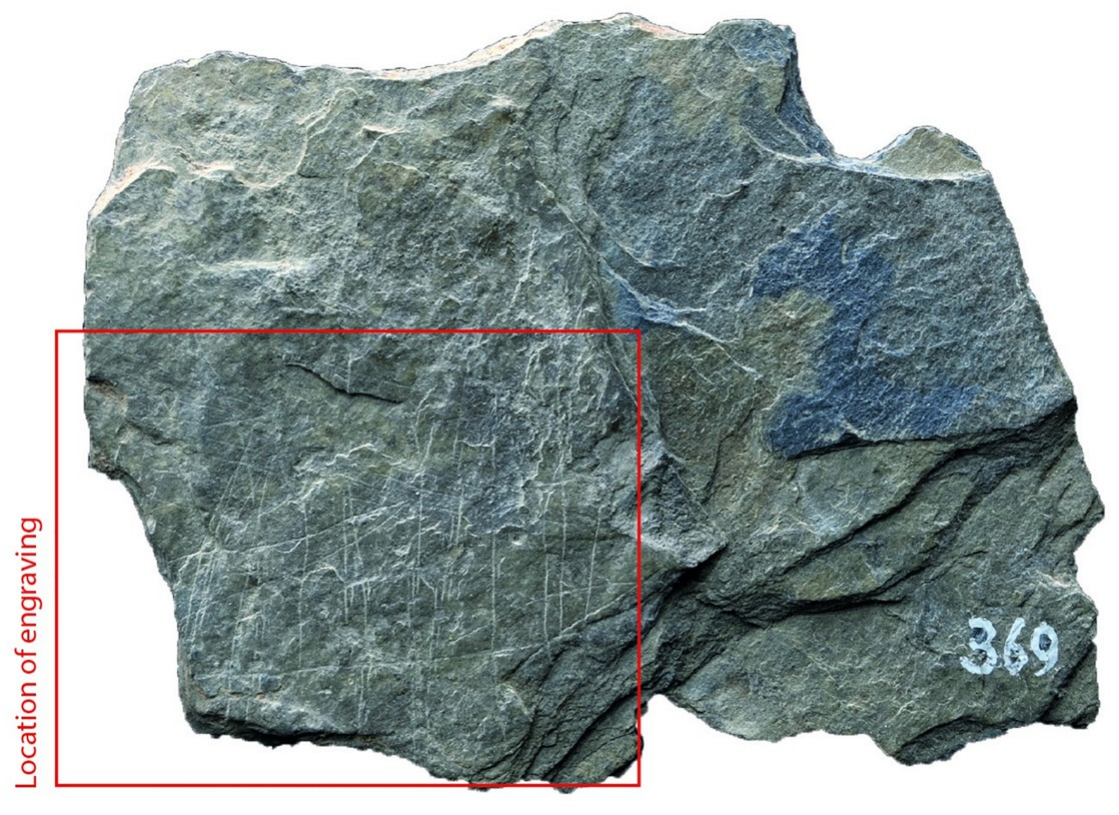
Plaquette 369, Gö 154 zp’’/380x. Dimensions of plaquette: 7.6cm (L) x 5.4cm (W) x 0.8cm (T).

**Fig 7:**
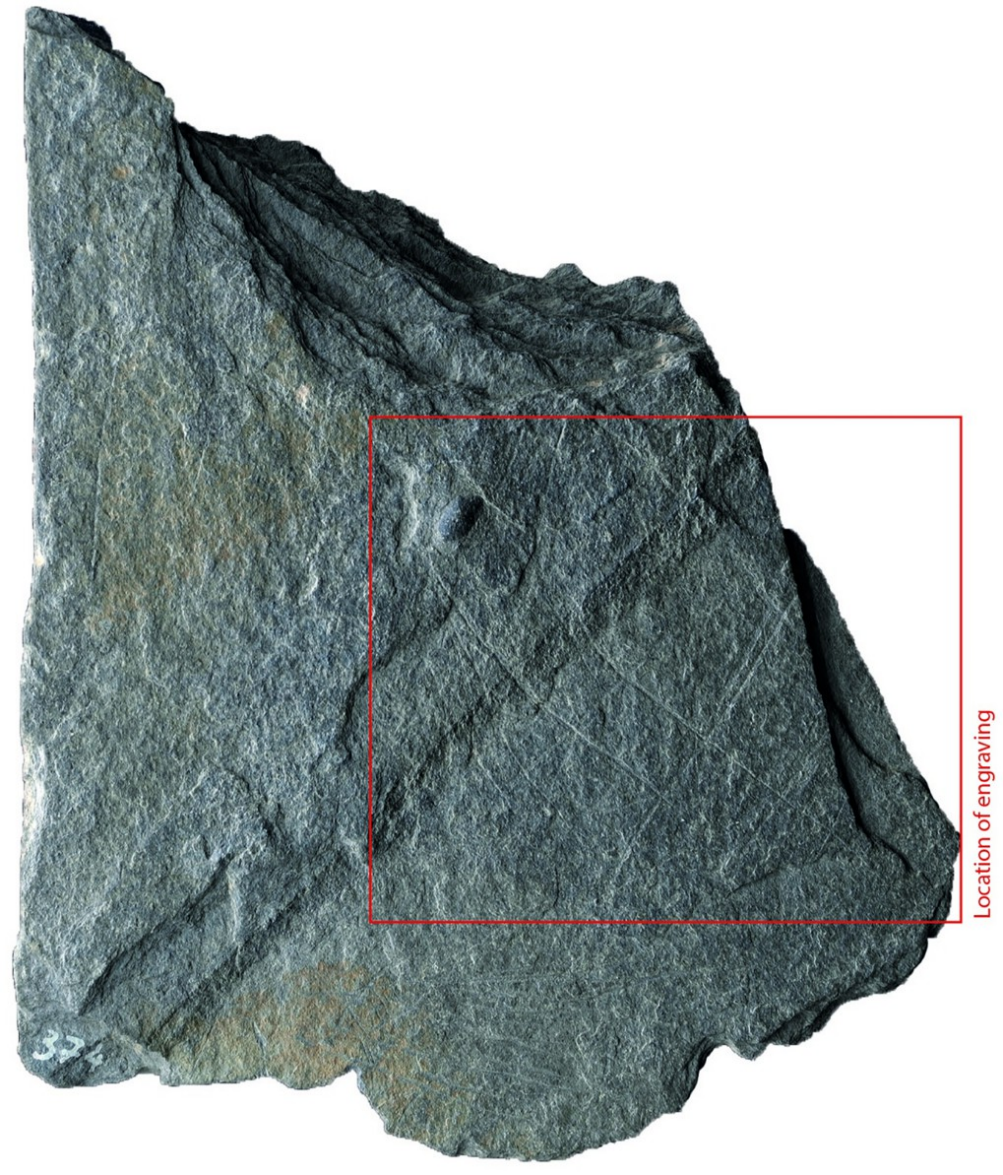
Plaquette 374, Gö 351:: PLI/85. Dimensions of plaquette: 11cm (L) x 12.5cm (W) x 1.6cm (T).

**Fig 8:**
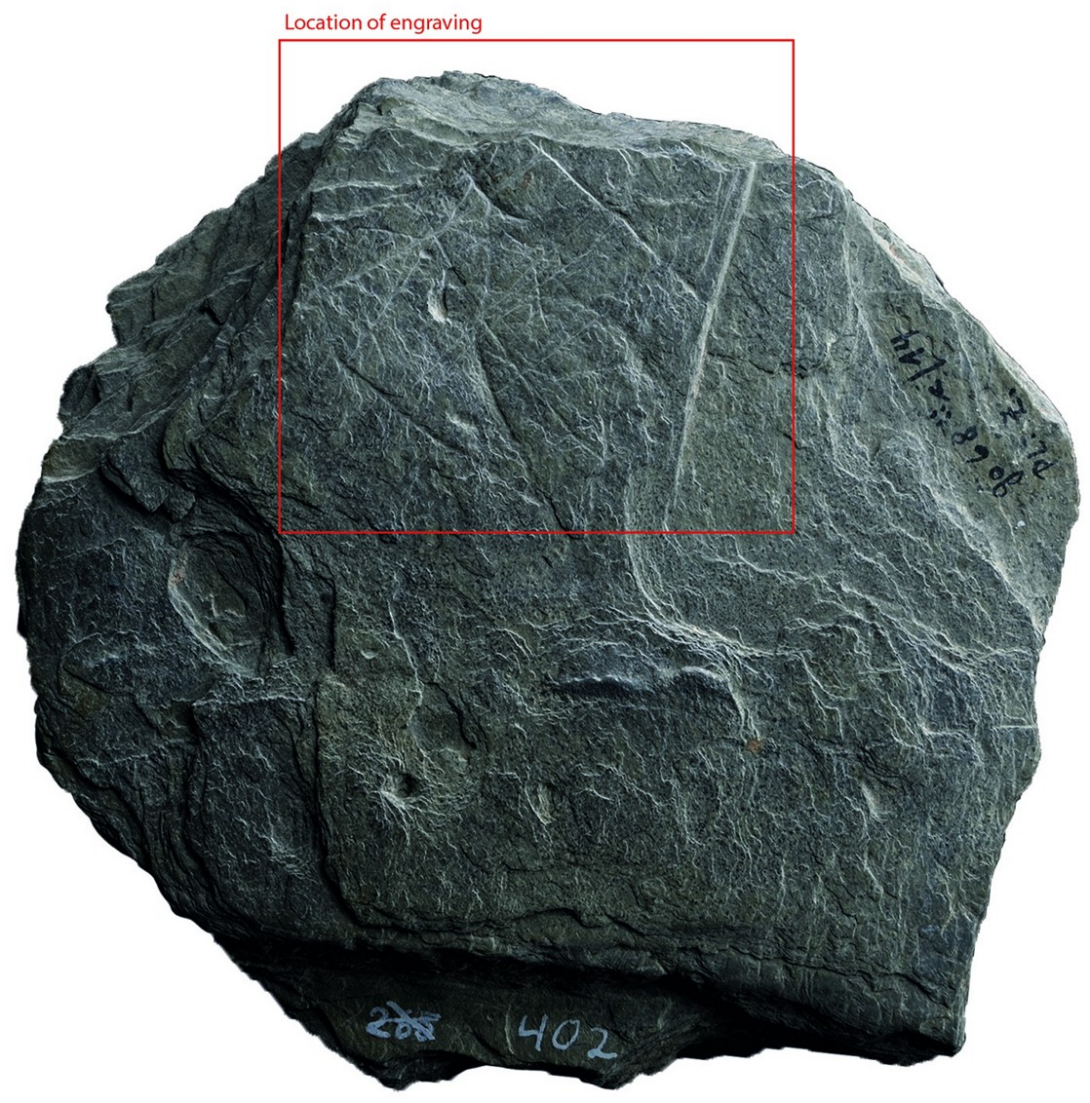
Plaquette 402, Gö 68:: c/14 Pl.I z. Dimensions of plaquette: 11cm (L) x 11cm (W) x 2.1cm (T).

All the depictions were meticulously engraved on flat and smooth areas of their host plaquettes. Shist plaquettes are natural fragments of rock that occurred in flat planes and which, therefore, are naturally flat. The plaquette surfaces had not been artificially smoothed ahead of the engravings; the engravings were created directly on the natural surface of the plaquettes, preferentially on their smoothest areas (Figs 1 to 8).

### Motif composition

A consistent feature across these plaquettes is the central motif of a fusiform within a grid (Figs 9 to 16). In all cases the fusiform shape was engraved first, followed by the grid pattern which was created to partially cover it (Figs 9 to 16; Table 2).

**Fig 9:**
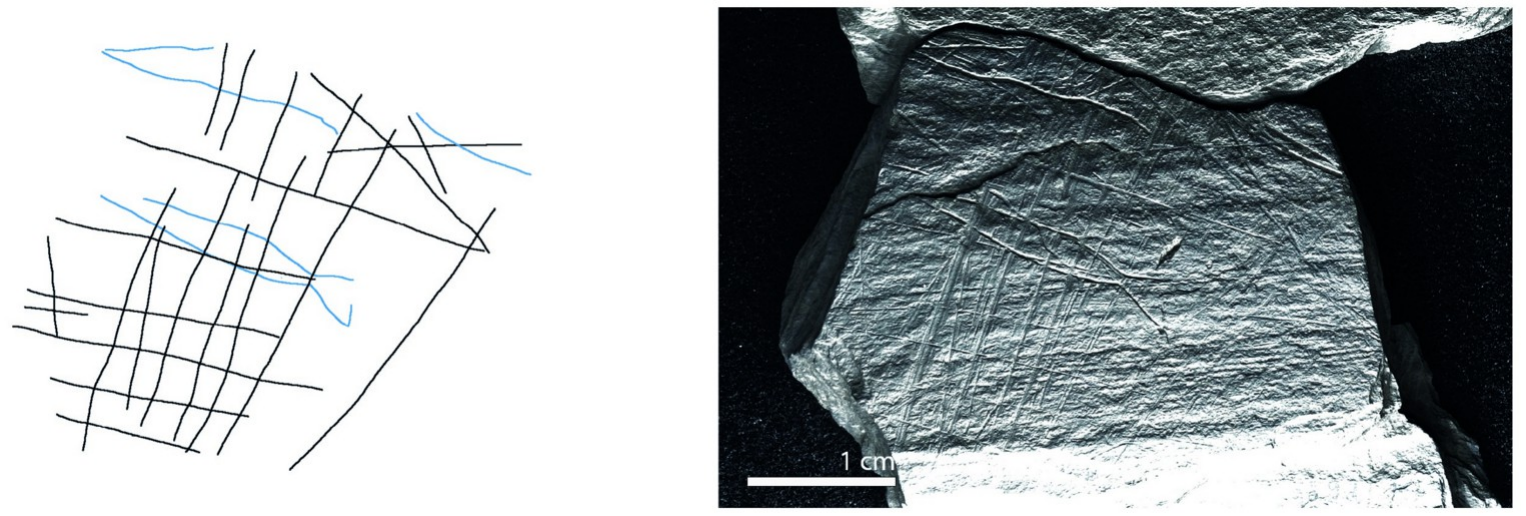
Plaquette 282, Gl 9:. d 117; Gö 50: d 160; Gö 50: d 106. Dimensions of engraving: 6cm (L) x 5cm (W); Location: Central part of a fragmented plaquette, flat surface; Fish: Fusiform shape, cranial, dorsal, and ventral sections, partial forked tail; Engraving: Fish first, followed by the net; fish at the center.

**Fig 10:**
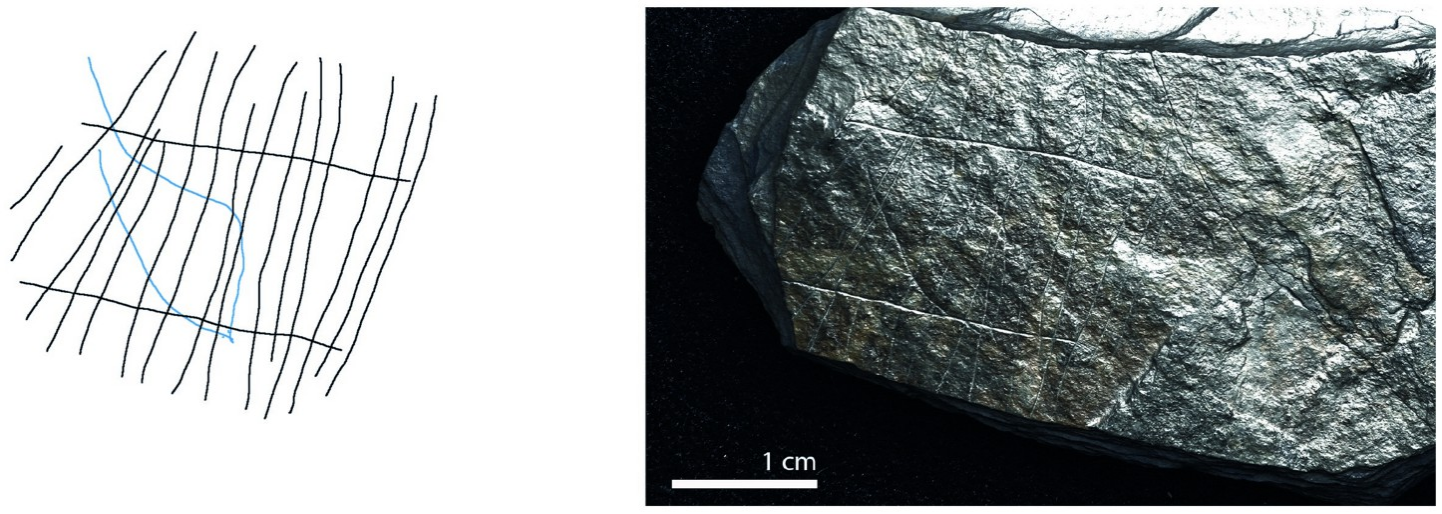
Plaquette 341, Gö 351::Pl.I/59. Dimensions of engraving: 6cm (L) x 5cm (W); Location: Left-hand side of the plaquette, flat surface; Fish: Fusiform shape, cranial, dorsal, and ventral sections, tail not visible; Engraving: Fish first, followed by the net; fish at the center.

**Fig 11:**
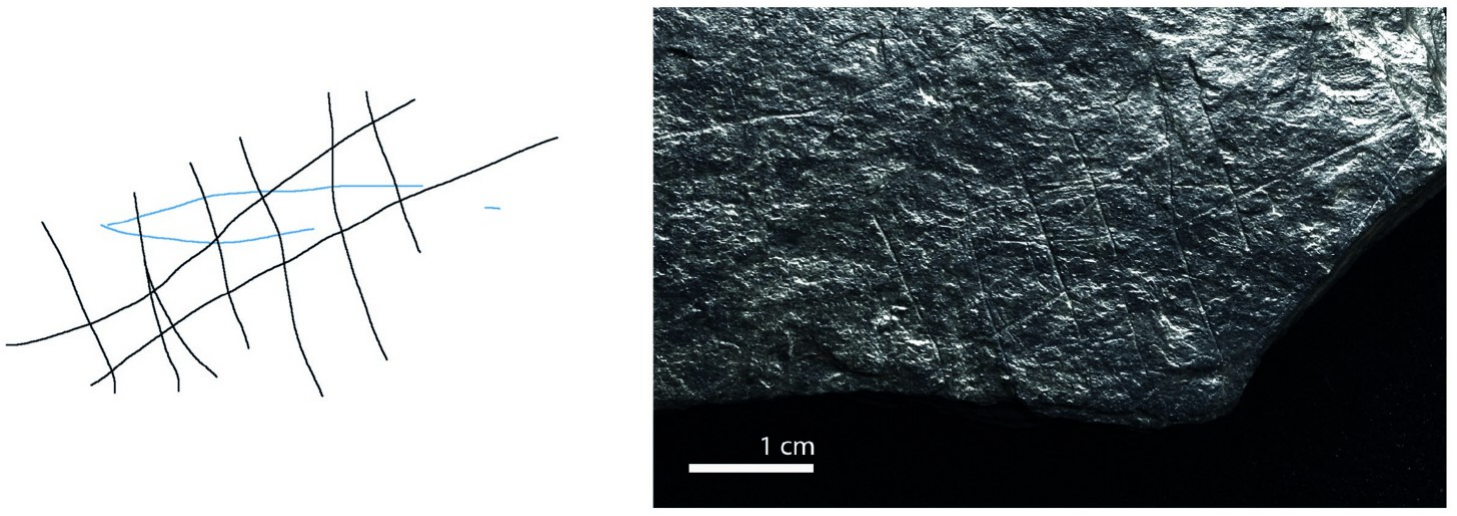
Plaquette 346, Gö 69’ c/46 Pl.I. Dimensions of engraving: 3cm (L) x 3cm (W); Location: Border of the plaquette, flat surface; Fish: Fusiform shape, cranial, dorsal, and ventral sections, interlaced with diamond-shaped meshes; Engraving: Fish first, followed by the net; fish at the center.

**Fig 12:**
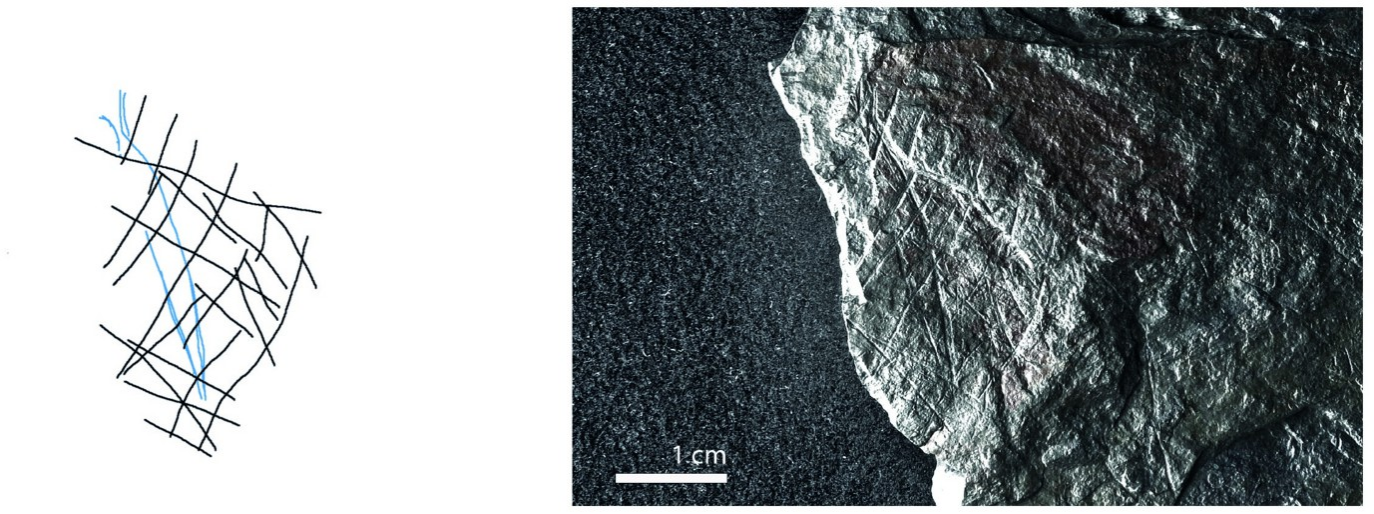
Plaquette 347, Gö 350:’ Pl.I 33; Gö 350:’ Pl.I 30. Dimensions of engraving: 3cm (L) x 2cm (W); Location: Edge of one plaquette fragment, flat surface; Fish: Fusiform shape, cranial, dorsal, and ventral sections, partial forked tail; Engraving: Fish first, followed by the net; fish at the center.

**Fig 13:**
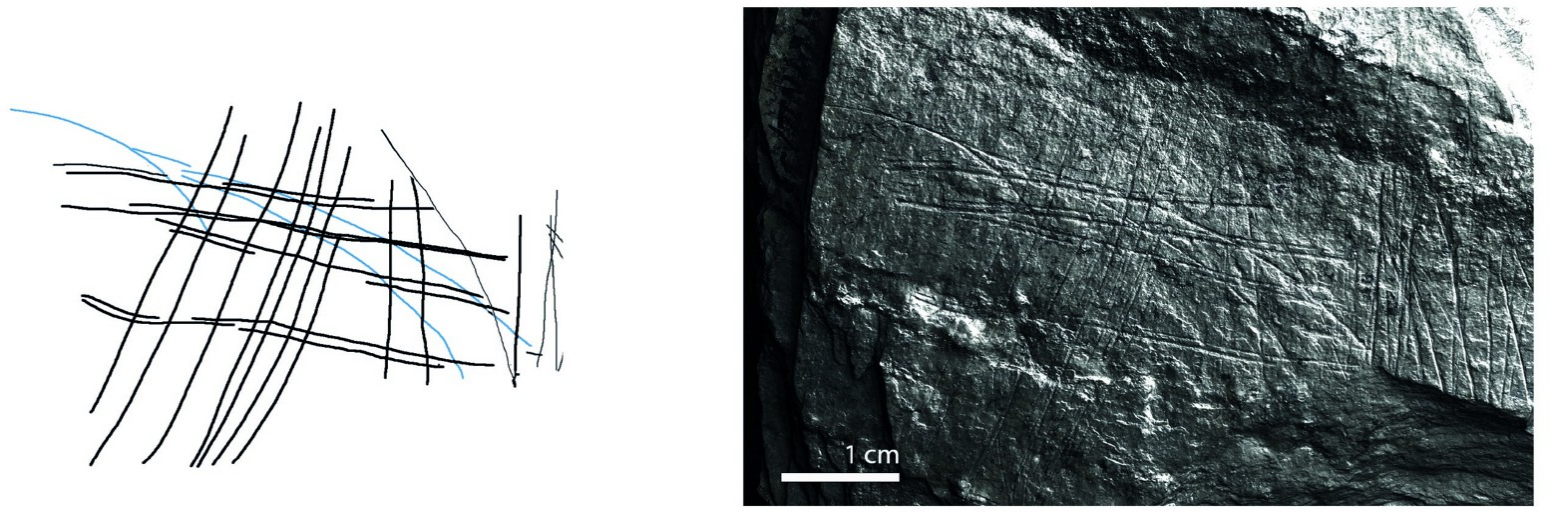
Plaquette 355, Gö 356 St67 N.II/351. Dimensions of engraving: 5cm (L) x 5cm (W); Location: Center of the plaquette, flat surface; Engraving: Fish first, followed by the net; fish at the center.

**Fig 14:**
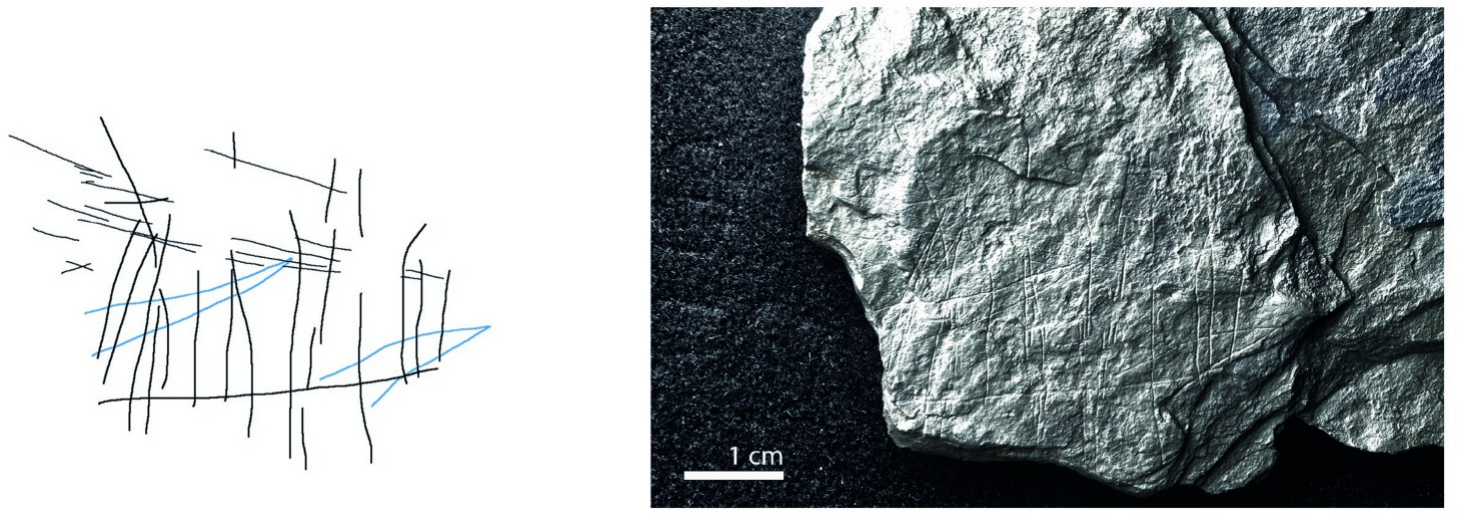
Plaquette 369, Gö 154 zp’’/380x. Dimensions of engraving: 4cm (L) x 4cm (W); Location: Left-hand side of the plaquette, flat surface; Fish: Fusiform shape, cranial, dorsal, and ventral sections; Engraving: Fish first, followed by the net; fish at the center.

**Fig 15:**
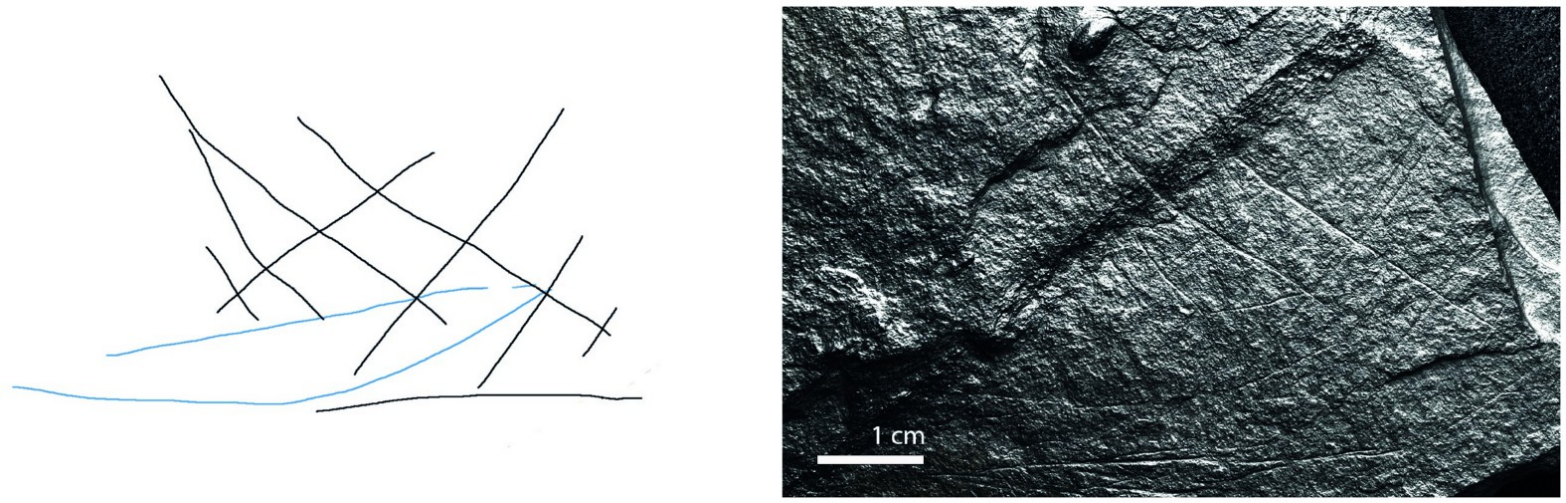
Plaquette 374, Gö 351:: PLI/85. Dimensions of engraving: 5cm (L) x 5cm (W); Location: Edge of the plaquette, flat surface; Fish: Fusiform shape, dorsal and ventral sections, interlaced with diamond-shaped meshes; Engraving: Fish first, followed by the net; fish at the center.

**Fig 16:**
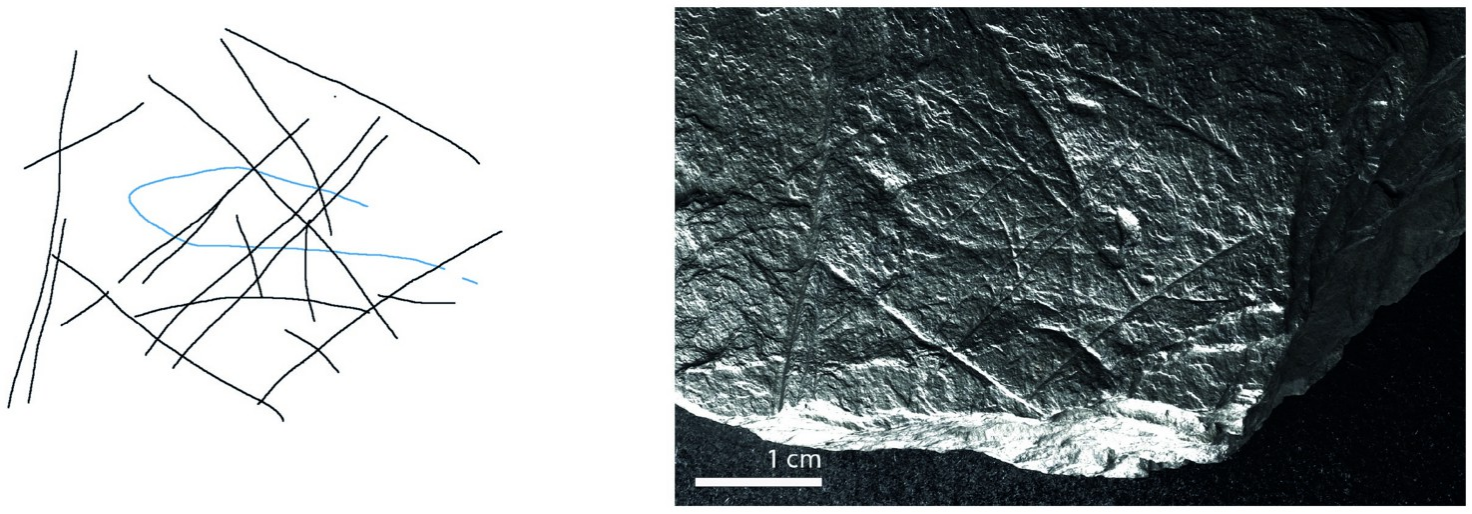
Plaquette 402, Gö 68:: c/14 Pl.I z. Dimensions of engraving: 4cm (L) x 5cm (W); Location: Edge of the plaquette, flat surface; Fish: Fusiform shape, dorsal and ventral sections, interlaced with diamond-shaped meshes; Engraving: Fish first, followed by the net; fish at the center.

**Table 2:** Placement and composition of fish motifs and grid patterns on plaquettes.

Plaquette number	Micro topography	Location of the engraving	Sequence of the engraving		Description of the engraving		Location of fish in the engraving
			First engraved	Second engraved	First engraved	Second engraved	
					Fish morphology	Grid description	
Gö 282 (Gö 9:. d 117)	Flat surface	Central part of the plaquette (engraved on a fragment). Original size 18L; 14.3W; 1,4T.	Fusiform	Grid	Fusiform shape, cranial, dorsal, and ventral sections, partial forked tail	Interlaced diamond-shaped mesh (approx. 8 vertical lines and 6 horizontal lines)	Fish at the center of the engraving
Gö 341	Flat surface	Edge of the plaquette	Fusiform	Grid	Fusiform shape, cranial, dorsal, and ventral sections	Interlaced square-shaped mesh (approx. 15 vertical line and 2 horizontal lines)	Fish at the center of the engraving
Gö 346	Flat surface	Edge of the plaquette	Fusiform	Grid	Fusiform shape, cranial, dorsal, and ventral sections	Interlaced diamond-shaped mesh (approx. 7 vertical lines and 2 horizontal lines)	Fish at the center of the engraving
Gö 347 (Gö 350:’ Pl.I 33)	Flat surface	Edge of the plaquette Original size 12.2L; 8.5W; 1T.	Fusiform	Grid	Fusiform shape, cranial, dorsal, and ventral sections, partial forked tail	Interlaced diamond-shaped mesh (approx. 7 vertical lines and 5 horizontal lines)	Fish at the center of the engraving
Gö 355	Flat surface	Center of the plaquette	Fusiform	Grid	Fusiform shape, cranial, dorsal, and ventral sections	Interlaced square-shaped mesh (approx. 9 vertical lines and 4 horizontal lines)	Fish at the center of the engraving
Gö 369	Flat surface	Edge of the plaquette	Fusiform	Grid	Fusiform shape, cranial, dorsal, and ventral sections	Interlaced square-shaped mesh (approx. 13 vertical lines and 3 horizontal lines)	Fish at the center of the engraving
Gö 374	Flat surface	Edge of the plaquette	Fusiform	Grid	Fusiform shape, dorsal and ventral sections	Interlaced diamond-shaped mesh (approx. 4 vertical lines and 4 horizontal lines)	Fish at the center of the engraving
Gö 402	Flat surface	Edge of the plaquette	Fusiform	Grid	Fusiform shape, dorsal and ventral sections	Interlaced diamond-shaped mesh (approx. 5 vertical lines and 4 horizontal lines)	Fish at the center of the engraving

Description and topographical data of fish engravings, including plaquette number, fish morphology, engraving location, detailed engraving features, grid pattern, fish position, and size sections.

### Motif placement

Fish-and-grid motifs were engraved at the edge of Plaquettes 341, 346, 347, 369, 374, and 402 (Figs 1 to 8; Table 2); and centrally on Plaquettes 282 and 355 (Figs 1 and 5; Table 2). had been Plaquettes 282 and 347 were previously broken, -and the fish and-grid engravings executed on fragments that refit with others (Plate 1). Plaquette 282 was broken into three pieces: Gö 9: d 117; Gö 50: d 160; and Gö 50: d 106. The fish and grid engraving was executed on fragment Gö 9: d117, in the centre of this fragment. Plaquette 347 was previously broken into two pieces: Gö 350:’ Pl.I 33 and Gö 350:’ Pl.I 30. The fish and grid engraving was executed on fragment Gö 350:’ Pl.I 33 after breakage. This is evidenced by the fact that the engraving has been made very close to the edge of this fragment, however, the grid lines do not continue on the other fragment but have stopped before the breakage line.

### Motif size

Generally, the engravings–both fish grids—are modest in size, grids measuring 2–3 cm by 5–6 cm in maximum dimension and fish 2–5 cm long and 0.25–0.8cm width. Fish occupy an area between 2% and 14% of their overlying grids; the grid being, therefore, consistently larger than the fish ‘contained’ within (Table 3).

**Table 3:** Comparative dimensions and proportions of fish motifs and grid patterns on plaquettes.

Plaquette number	Motif dimensions				
	Grid dimensions (cm)		Fusiform dimensions (cm)		Fish representation inside the grid (%)
	Length	Width	Length	Width	
Gö 282 (Gö 9:. d 117)	6	5	2	0.3	2%
Gö 341	6	5	2	0.8	5.33%
Gö 346	3	3	2.5	0.4	11.11%
Gö 347 (Gö 350:’ Pl.I 33)	3	2	3	0.25	12.50%
Gö 355	5	5	3	0.3	3.60%
Gö 369	4	4	2	0.3	3.75%
Gö 374	5	5	5	0.7	14%
Gö 402	4	5	2	0.6	7%

Detailed analysis of fish motif engravings, including plaquette number, fish morphology, engraving location, description, grid pattern, fish position, and size sections.

### Anatomical details

The compositions include discernible cranial (head), dorsal (back), and ventral (belly) parts of fish, providing clear anatomical distinctions. Some of them also include a fish tail (e.g. Plaquettes 282 and 347). Overall, however, the fish depictions are characterized by abstraction and minimalism.

### Grid description

The grid lines—formed by 4 to 15 vertical lines and by 2 to 6 horizontal lines (Table 2)—have similar form to those of the fish, and appear, therefore, to have been made by the same tool and, we assume, at the same time. The spacing between the vertical lines and the spacing between the horizontal lines of the grid is uneven but allows the fish motifs to remain visible behind the lines. The grid pattern.

## Discussion

The use of RTI technology in the analysis of Plaquettes 341, 346, 347, 355, 369, 374, and 402 played a critical role in identifying the previously unnoticed fish-and-grid engravings. It was pivotal in enabling us to discern that these grid and fish depictions are meaningfully connected, similarly layered (created in the same sequence), and engraved in one instance, almost certainly with the same tool (and we assume–although cannot demonstrate–by the same person. Analysis under RTI Visualisation modes revealed that in all instances, the fish motifs were engraved first, and were subsequently overlaid with the grid lines (Figs 9 and 10), thus framing the fish motifs as the central element of the overall motif (Table 3; Figs 9 to 16).

The dimensions of the grid are particularly significant in relation to the fish motifs. Considering the measurements (Figs 9 to 16) of the engravings of fish and grid on various plaquettes, it is evident that the grid patterns occupy a (marginally) larger area (Table 3; Figs 9 to 16) than the fish. This relationship between the size of the grid and the fish motifs (Table 3) underscores the importance of spatial composition in these engravings, as the grid lines not only frame but also interact with (perhaps contain’) the central fish. Thus, the central location of the fish, within grid lines which clearly overlay them and which h exceed them in size, together suggest that the grids represent a form of container—a net or trap–into which the fish have become enmeshed. The sequence of these engravings, adhering to this specific order where the fish motifs are engraved first, followed by the grid motifs, underscores a deliberate artistic process and design, strongly suggesting the depiction of an animal that was at first free swimming (when alone), which was then captured by the creation of the grid.

This combination of motifs (fish and grid) must be read as a scenic depiction, which in any form are only rarely observed in the Gönnersdorf corpus. The schematic, headless, and highly-stylised human female depictions for which the site is famous [56, 57] occasionally appear in scenes implying the act of meeting or gathering (perhaps dancing) of some sort [61]. Additionally, a singular scenic depiction involves a running horse with birds moving under its belly (Plaquette 168) clearly emphasising the running of the horse in a landscape rather than simply the depiction of the horse itself [54].

Animal depictions from Gönnersdorf, in contrast to those of human females, are usually characterized by a high degree of naturalism, which is typical for the Late Magdalenian art of the region [52]. The fish-and-grid scenes, however, are characterized by a minimalist and abstract style, with elongated and streamlined shapes focusing only on their essential forms and avoiding extraneous details. We may see this as a deliberate simplification a focus on essential geometric forms that is also observed in other late Magdalenian sites, indicating a wider trend towards reductionism and abstraction in specific contexts of animal representation [62, 63]. From an art-history perspective, these depictions can be clearly identified as "schematic art," where the focus is on simplification rather than detailed realism., which grows in importance during the succeeding epipalaeolithic (Guy 1993), reflecting the deliberate artistic choice to emphasize geometric forms like ellipses, ovals, and fusiform outlines [64]. Except for two sagittiform fish which feature symmetrical forked tails (Plaquettes 282 and 347), adding a distinct touch within this minimalist style, a notable characteristic of all fish engravings is the absence of detail in the representation of both the ventral and dorsal parts of the fish. These artistic choices mirror trends observed in other European Pleistocene sites, such as Valcamonica, where fish representations also exhibit minimalist traits and are often depicted in conjunction with geometric trap-like structures. It has been thought that this reflect a symbolic rather than naturalistic importance of these motifs [44]. They certainly distinguish these motifs from the rich corpus of detailed and naturalistic depictions of other animal species at Gönnersdorf and elsewhere [53–55].

Lorblanchet (1993) commenting on the artistic representation of fish in Palaeolithic parietal art, notes that while these depictions are not anatomically detailed, their general form often contains enough detail to facilitate identification [65]. The artistic techniques employed for these depictions were as diverse as those used for other figurative species, including relief, engraving, and painting, suggesting that in this sense at least they formed part of a wider artistic repertoire, but the simplicity of the Gönnersdorf fish depictions, coupled with the dominant depiction of traps/nets alongside the fish, suggests that the focus of these engravings was not on the fish as a subject per se, but rather on the action of fishing, through the use of nets.

Fishing activities were suggested by the presence of fish remains at Gönnersdorf [5]. Subsequently, Street and Turner (2013) [6], subsequently revealed evidence of fish consumption at the site. In both Sector KI and Sector KIII, a total of 21 fish remains were recovered, although a detailed size comparison of these fish bones have not yet been published. Fishing activities in the Palaeolithic period were likely influenced by seasonal variations, as fish tend to migrate, spawn, or become more abundant at certain times of the year. Nets would most likely have been the ideal equipment for catching larger quantities of fish during migrating periods.

Fishing nets in the Upper Palaeolithic may be indirectly evidenced by vegetal textile impressions found at the Mid Upper Palaeolithic sites ofof Dolni Vestonice and Pavlov I in Moravia, Czech Republic [66–69]. These imprints reveal advanced textile and cordage techniques dating back to approximately 30,000 cal. B.P. The presence of knotted cordage impressions specifically suggests the use of netting fragments, although of course this may have had wider or other uses than for fishing [68]. Additionally, the discovery of a fragment of rope in Lascaux cave (Dordogne, France), indicates the presence of rope/netting technology ~21,500 cal. BP [70–73].

During the Magdalenian, fishing nets were likely used to target specific fish sizes. At sites like Gare de Conduché cave and Ste Eulalie cave in France, evidence suggests deliberate fishing practices with nets designed specifically to capture larger fish [65, 74].

Pollen analysis at Palaeolithic sites such as Dolní Věstonice I, II, and Pavlov I suggests an abundance of plant species suitable for textile, basketry, and cordage production [66, 67, 69]. Notable plants like alder and yew with fibrous bark were present, along with milkweed and nettle, historically used for perishable goods manufacture [75, 76]. Iconographic representations on Upper Palaeolithic Venus figurines, such as the Gönnersdorf Plaquette 51, depict clothing, suggesting textile use [71, 77]. These textiles were likely produced using basic tools like shuttles, spacers, and awls. Gönnersdorf has provided further evidence, including bone and antler inventories displaying cordage impressions and bone needles associated with textile production [78, 79].

The findings from Gönnersdorf highlight the complexity and ingenuity of Magdalenian fishing practices, as revealed through the detailed analysis of the plaquettes. The integration of grid motifs with fish depictions may suggest a deliberate artistic and functional approach, where fishing nets were not only tools for survival but potentially significant elements in the symbolic and cultural landscape of the time. The minimalist representation of these scenes, in contrast to more naturalistic animal depictions, could indicate a focused narrative on the act of fishing itself, emphasizing the technological and social aspects of this activity: perhaps the fish remain vague–depicted in outline and with little detail—as that is how they looked while under water, or that their subsequent processing required relatively little attention to detail, at least in comparison to the butchery of large terrestrial game. By contextualizing these findings within broader Palaeolithic art and subsistence strategies, we may gain a deeper appreciation of the role that fishing played in the daily lives and collective identities of Magdalenian communities. Were fish conceived of as vague, small, gatherable resources, at least when obtained by indirect (netting) techniques? We need further research into how Upper Palaeolithic hunter-gatherer communities might have adapted their artistic expression to reflect their interactions with and perceptions of their resource environment, particularly in the context of resource exploitation and cultural symbolism.

## Conclusions

The Gönnersdorf engravings provide valuable insights into the fishing techniques and tools used by Palaeolithic peoples, and how these practices were translated into visual culture through the depiction of nets characterized by interlaced diamond-shaped and square meshes. The minimalist artistic style of the fish engravings, combined with the intricate representation of nets, emphasizes the action of fishing rather than merely depicting fish themselves. This interpretation is further reinforced by the artistic choices in canvas size, motif details, and engraving sequence, which collectively highlight the deliberate and sophisticated approach to capturing and handling fish during the Magdalenian period. Overall, these depictions contribute significantly to our understanding of Palaeolithic fishing practices and the cultural expressions of the Magdalenian people.

Importantly, the presence of fish remains at Gönnersdorf, the evidence for textile manufacture and use, along with the depiction of fish within nets on eight engraved plaquettes, provide the first unambiguous evidence for net fishing in a Magdalenian context.

Our comparative analysis including other archaeological sites from the same period, such as Altamira and Lascaux, highlights Gönnersdorf’s contribution to our understanding of Palaeolithic art and subsistence strategies. Unlike the more celebrated sites known for their vivid fish depictions, Gönnersdorf’s abstract and minimalist style offers a fresh perspective on the socio-cultural dynamics of Magdalenian communities. Fishing with nets, deriving from a broad spectrum economy, reveals the diversity, adaptability and creativity of prehistoric communities, showcasing their proficiency in utilizing a variety of fishing techniques to sustainably exploit aquatic resources. This research not only enhances our understanding of the diverse subsistence strategies of Palaeolithic societies but also contributes to the broader discourse on the complexity and richness of their cultural practices. By placing a spotlight on the often-overlooked aspect of the practice of fishing and the representation of that practice in art, our study adds to a more comprehensive and dynamic picture of subsistence during the Upper Palaeolithicopening new pathways for future research in this field. Evidently fishing played a more significant role in shaping social and cultural practices than previously recognized, as evidenced by the aggregation patterns around bountiful fishing sites, and is therefore a subject matter worth of further investigation.
